# Health-Related Predictors of Quality of Life in Cancer Patients in Saudi Arabia

**DOI:** 10.1007/s13187-017-1198-3

**Published:** 2017-03-07

**Authors:** Anwar E. Ahmed, Alaa S. Almuzaini, Mohannad A. Alsadhan, Abdulrahman G. Alharbi, Hanin S. Almuzaini, Yosra Z. Ali, Abdul-Rahman Jazieh

**Affiliations:** 1 King Abdullah International Medical Research Center (KAIMRC), Riyadh, Saudi Arabia; 2 King Saud bin Abdulaziz University for Health Sciences, Riyadh, Saudi Arabia; 3 College of Medicine, King Saud University, Riyadh, Saudi Arabia; 4 College of Medicine, Imam Abdulrahman Al Faisal University, Dammam, Saudi Arabia; 5 Department of Oncology, King Abdulaziz Medical City, Riyadh, Saudi Arabia

**Keywords:** SF-36, QoL, Regular exercise, First-year-cancer diagnosis, Saudi Arabia

## Abstract

Research on Saudi Arabian cancer patients is a priority at King Abdulaziz Medical City (KAMC), Riyadh, Saudi Arabia. Because there is limited research on the quality of life (QoL) of Saudi Arabian cancer patients, the aim of this study was to identify the predictors of the QoL in a sample of Saudis with cancer. In August 2016, a cross-sectional study was conducted on 438 patients with a variety of cancer types (145 breast, 109 colorectal, 38 leukemia, 45 lymphoma, and 99 other types) who attended the Oncology Outpatient Clinics at KAMC. Sociodemographics, clinical symptoms, and cancer treatments were collected for each patient. We used the SF-36 instrument to assess QoL. Of the cancer patients studied, 28.4% had a family history of cancer, and, according to subgroup analyses, the elderly, those lacking formal education, the unemployed, those diagnosed with Stage III/IV, and those with metastasis had significantly worse physical functions than the other cancer patients. According to multiple linear regression analyses, cancer patients who exercised regularly tended to have better physical function, emotional role function, vitality, social function, and general health (increase in SF-36 scores of 8.82, 9.75, 5.54, 6.66, and 4.97, respectively). Patients with first-year-after-cancer diagnosis tended to have poor emotional wellbeing, social function, and general health (decrease in SF-36 scores of 5.20, 7.34, and 6.12, respectively). Newly diagnosed cancer patients and patients who did not exercise tended to experience significantly poor QoL in several domains; thus, the effectiveness of exercise must be assessed in Saudi cancer patients as an intervention to improve QoL.

## Introduction

According to the Saudi Ministry of Health Cancer Registry in Riyadh, more than 15,653 people in Saudi Arabia (77.6% were Saudis) were diagnosed with cancer in 2013. The crude incidence rate was 57.5 per 100,000 population per year. The Saudi Government’s vision for 2030 is to significantly mitigate the challenges faced by the health sector in preventing cancers through analyzing independent risk factors and improving health and control cancer outcomes through treating the symptoms of cancers.

Cancer not only affects patients physically, but it may also impact the quality of life (QoL) of cancer survivors negatively [1, 2]. Recently, much attention has been paid to the negative impact of cancer and its treatment on the QoL in cancer patients. Several reports have indicated that greater QoL impairment in patients with cancer may be attributable to treatment side effects, cancer symptoms, and psychological distress [2–4].

There is evidence that older age has negative effects on the QoL in cancer patients [5, 6], while gender has an influence on the degree of QoL impairment [7]. Lack of education has a negative effect on cancer patients [7], and low income has also been negatively associated with QoL in cancer patients [8–11]. Other factors contributing to QoL impairment may include clinical presentations of cancer patients such as the stage, type, and site of the cancer [7, 12]. It has been documented internationally that measuring the quality of life in cancer patients is an important aspect of cancer management and treatment, and could serve as an effective tool for clinical trials [1, 13–15].

To date, research on QoL in cancer patients in Saudi Arabia has been insufficient. Only three studies in Saudi Arabia have addressed the quality of life in cancer patients. Colorectal cancer [16] and breast cancer [17, 18] patients were reported to have a low QoL. According to the authors, there are numerous factors associated with a major reduction in all domains of QoL, including educational level, employment status, pathological staging, and tumor location [16–18]. There are numerous self-report questionnaires used to measure QoL, including the European Organization for Research and Treatment of Cancer (EORTC) quality of life [19] which is used by Almutairi et al. The Short-Form Health Survey SF-36 (the RAND 36-item) questionnaire [20] is a self-report questionnaire commonly used to assess QoL, and it has been used consistently in Saudi patients with sickle cell disease [21, 22]. However, there is a paucity of data using SF-36 measure in Saudi cancer patients.

The impact of sociodemographics, cancer characteristics, and treatment are important to consider when assessing QoL in the cancer population. It allows clinicians to describe and assess the health status of cancer patients, provide interventions, and measure their effectiveness. This study is of interest to oncologists who provide routine care to cancer patients in Saudi Arabia. Research on Saudi Arabian cancer patients is a priority at King Abdulaziz Medical City (KAMC), Riyadh, Saudi Arabia. In this study, we used data from a study conducted at King Abdulaziz Medical City in Riyadh (KAMC-R) to determine the impact of sociodemographics, clinical symptoms, and cancer treatments on QoL measures in Saudi cancer patients. We hypothesized that being elderly, newly diagnosed patients, and the cancer prognosis would have a negative impact on QoL in Saudi cancer patients. We also hypothesized that exercise may impact QoL positively in cancer patients.

## Materials and Methods

A survey study was conducted in the outpatient oncology clinics, KAMC, Ministry of National Guard Health Affairs. The study obtained scientific and ethical approval from the IRB office at King Abdullah International Medical Research Center (KAIMRC), Riyadh (# RSS16/004). The study included a consecutive sample of cancer patients with different types of cancer who were attending outpatient oncology clinics for follow-up with oncology specialists during the study period (August 14–31/2016). The subjects of the study administered a one-time survey with a consent form explaining the aims of the study and asking whether they wanted to complete the survey. Assent was obtained from parents of all cancer patients with ages between 14 and 17 years. We obtained written consent from those patients age 18 years and above.

A total of 540 subjects who consented were administered the survey, and 436 surveys were completed and returned (145 breast, 45 lymphoma, 109 colorectal, 38 leukemia, and 99 other types of cancer) with a response rate of 80.7%. Sociodemographics data were collected for each patient such as age, gender, height, weight, university degree, marital status, regular exercise, family support, and employment status. We collected clinical data on patients and their cancer characteristics such as type of cancer, family history of cancer, cancer stage (I, II, III, or IV), multiple tumors, newly diagnosed cancer patients or first-year-after-cancer diagnosis, whether patient received chemotherapy, whether patient had surgery to remove tumors, whether patient received immunotherapy, whether patient received radiation therapy antibiotics, side effects of treatment, metastasis, sleep deprivation, fever, and chronic disease other than cancer. The following section describes the quality of life questionnaire used to assess patients’ health status.

### Quality of Life Instrument

Quality of life was assessed by the Medical Outcomes Study 36-item short-form (SF-36) questionnaire [20], an instrument with an Arabic version [23] and established reliability that provides subjective evaluation of quality of life. It has been used in general and disease-specific populations. The SF-36 is a self-rated tool comprising 36 items grouped into eight domains: physical function, physical role health, emotional role functions, vitality, emotional wellbeing, social function, bodily pain, and general health. Each of these domains ranges from 0 (poor health) to 100 (best health). The SF-36 questionnaire was found to be reliable in this population with Cronbach’s alpha values ranging between 0.60 “social function” and 0.91 “physical function.”

### Data Analysis

**Fig. 1:**
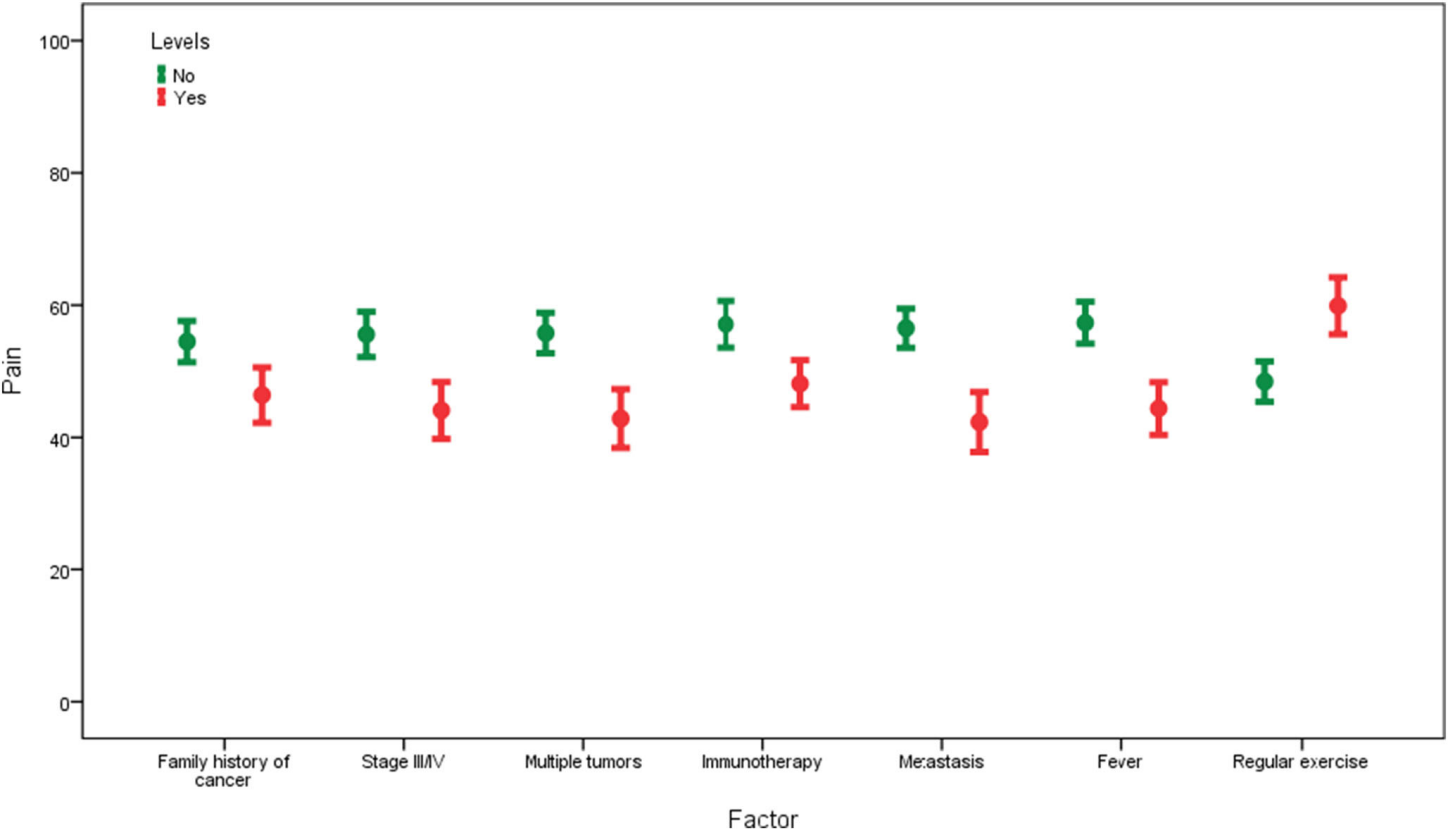
Error bar chart: impact of cancer complications on bodily pain ratings. Notes: The higher the score, the less pain

The data analysis was conducted using IBM SPSS Statistics 23 (SPSS, Chicago, IL). *Patients’ characteristics*: sample statistics such as means and standard deviation were used to summarize numerical data. Counts and percentages were used to summarize categorical data (Table 1). *Bivariate analyses*: In order to account for 24 multiple comparisons, the Bonferroni correction of *α*/*n* = 0.05/24 = 0.0021 was used to compare QoL differences between sociodemographics and clinical characteristics (Tables 1 and 2, Fig. 1). *Multivariate analyses*: Multiple linear regression models were used to examine the relationship between the sociodemographics, clinical symptoms, and cancer treatments and each QoL domain, and to identify predictors of the SF-36 subscales. Regression coefficients were used to interpret the linear regression findings. In all multivariate analyses, the significance level (*α*) was set at 0.05.

**Table 1:** Differences in quality of life by sociodemographics and clinical characteristics (*N* = 436)

		Overall		Physical functioning			Role limitations due to physical health			Role limitations due to emotional problems			Vitality		
Characteristics		*n*	%	Mean	SD	*P*	Mean	SD	*P*	Mean	SD	*P*	Mean	SD	*P*
Gender	Male	157	36.0	46.8	30.8	0.429	24.7	36.6	0.620	33.3	41.9	0.507	44.0	21.6	0.924
	Female	279	64.0	49.1	28.7		26.5	37.6		30.6	41.2		43.8	22.3	
Elderly	No	270	61.9	55.1	27.6	0.001*	25.8	37.3	0.985	32.1	41.0	0.737	46.1	22.0	0.009
	Yes	166	38.1	37.2	29.0		25.9	37.1		30.7	42.2		40.4	21.7	
University	No	312	71.6	44.1	28.8	0.001*	25.9	37.0	0.985	29.9	40.6	0.185	42.2	22.1	0.010
	Yes	124	28.4	58.9	28.4		25.8	37.8		35.8	43.4		48.2	21.2	
Employed	No	321	73.6	45.6	28.3	0.001*	26.5	37.0	0.562	31.5	41.5	0.926	43.2	21.8	0.246
	Yes	115	26.4	56.0	31.2		24.1	37.7		31.9	41.5		46.0	22.4	
Married	No	99	22.7	47.2	30.1	0.678	30.3	38.8	0.177	32.0	40.1	0.911	45.2	23.6	0.524
	Yes	337	77.3	48.6	29.3		24.6	36.6		31.5	41.9		43.5	21.5	
Obese	No	273	71.7	50.1	29.2	0.217	28.0	38.9	0.216	32.8	42.3	0.460	44.4	21.6	0.876
	Yes	108	28.3	45.9	31.3		22.9	35.0		29.3	40.9		44.0	23.7	
Family history of cancer	No	312	71.6	47.5	29.7	0.339	27.6	37.9	0.112	33.3	42.3	0.147	44.9	22.4	0.129
	Yes	124	28.4	50.4	28.6		21.4	35.0		27.2	39.0		41.4	20.7	
1st year after cancer diagnosis	No	196	45.1	46.9	28.1	0.405	21.8	33.7	0.045	32.0	41.5	0.800	42.5	23.3	0.243
	Yes	239	54.9	49.2	30.4		28.9	39.4		31.0	41.3		45.0	20.8	
Cancer Types	Breast	145	33.3	54.4	28.8	0.002*	29.3	38.3	0.292	33.6	42.2	0.137	43.9	20.8	0.242
	Colorectal	109	25.0	42.7	26.6		23.4	35.1		32.1	41.6		41.2	22.4	
	Leukemia	38	8.7	52.9	29.5		23.7	37.2		23.7	38.7		42.1	23.4	
	Lymphoma	45	10.3	52.8	29.7		32.8	39.8		43.0	42.4		50.0	24.8	
	Others	99	22.7	41.7	31.2		21.2	36.3		25.9	40.0		44.8	21.2	
Stage III/IV	I/II	216	58.7	53.1	29.4	0.001*	29.2	38.3	0.006	36.0	43.2	0.001*	47.4	20.3	0.001*
	III/IV	152	41.3	42.0	29.1		18.9	32.9		22.1	36.2		38.6	22.5	
Multiple tumors	No	291	68.5	49.9	29.6	0.034	28.7	38.5	0.003	35.9	42.8	0.001*	46.5	21.7	0.001*
	Yes	134	31.5	43.4	28.9		18.1	31.8		22.6	36.9		37.5	21.4	
Cancer surgery	No	199	45.6	48.1	30.5	0.890	25.0	37.3	0.659	30.5	41.0	0.615	43.6	21.3	0.800
	Yes	237	54.4	48.5	28.6		26.6	37.2		32.5	41.8		44.2	22.6	
Chemotherapy	No	101	23.2	48.2	29.8	0.958	33.7	41.3	0.027	38.3	45.1	0.082	46.6	22.5	0.164
	Yes	335	76.8	48.3	29.4		23.5	35.6		29.6	40.1		43.1	21.8	
Radiation therapy	No	238	54.7	50.3	29.8	0.142	29.0	39.3	0.055	33.3	41.8	0.352	46.4	22.6	0.009
	Yes	197	45.3	46.1	28.7		22.2	34.2		29.6	41.1		40.9	20.9	
Immunotherapy	No	206	47.5	51.3	30.7	0.054	28.5	40.3	0.149	37.1	44.3	0.010	47.0	21.2	0.007
	Yes	228	52.5	45.9	27.9		23.4	34.0		26.8	38.2		41.3	22.3	
Antibodies	No	319	75.2	49.9	29.1	0.115	26.8	38.3	0.477	34.6	43.0	0.013	45.2	22.4	0.123
	Yes	105	24.8	44.6	31.1		23.8	34.4		23.8	36.3		41.3	20.8	
Metastasis	No	303	69.5	51.2	29.0	0.002*	28.5	38.5	0.016	35.1	42.9	0.005	46.9	21.7	0.001*
	Yes	133	30.5	41.8	29.5		19.7	33.4		23.6	36.9		37.2	21.3	
Fever	No	263	60.3	50.4	30.7	0.058	30.1	39.5	0.002*	38.1	43.9	0.001*	46.8	22.1	0.001*
	Yes	173	39.7	45.1	27.1		19.4	32.4		21.6	35.2		39.6	21.1	
Family support	No	37	8.5	49.9	33.0	0.736	26.4	35.3	0.933	42.3	45.6	0.137	43.4	25.7	0.878
	Yes	399	91.5	48.2	29.1		25.8	37.4		30.6	40.9		44.0	21.7	
Chronic disease other than cancer	No	271	62.2	52.0	29.1	0.001*	25.9	38.4	0.964	29.4	40.7	0.160	44.7	21.7	0.358
	Yes	165	37.8	42.2	29.0		25.8	35.3		35.2	42.5		42.7	22.5	
Regular exercise	No	293	67.2	44.4	29.6	0.001*	23.2	36.1	0.038	28.0	41.0	0.009	41.1	21.6	0.001*
	Yes	143	32.8	56.2	27.4		31.3	39.0		38.9	41.5		49.7	21.7	

*The variable is significant using Bonferroni correction cut-off at *α*/*n* = 0.05/24 = 0.0021, where *n* is the number of tests, *P*=P-value.

**Table 2:** Differences in quality of life by sociodemographics and clinical characteristics (*N* = 436)

		Emotional wellbeing			Social functioning			Pain			General health		
Characteristics		Mean	SD	*P*	Mean	SD	*P*	Mean	SD	*P*	Mean	SD	*P*
Gender	Male	63.7	19.7	0.479	55.7	26.8	0.470	56.0	27.0	0.020	51.7	16.7	0.528
	Female	62.3	20.4		57.7	27.7		49.7	26.8		50.5	18.3	
Elderly	No	62.1	20.9	0.374	58.9	27.2	0.065	51.4	27.7	0.557	52.2	18.0	0.054
	Yes	63.9	18.9		53.9	27.4		52.9	25.9		48.9	17.1	
University	No	62.2	19.9	0.344	56.8	27.8	0.786	50.2	27.3	0.030	49.6	17.9	0.009
	Yes	64.3	20.7		57.6	26.2		56.4	26.0		54.4	17.0	
Employed	No	62.8	19.6	0.978	57.7	26.6	0.389	51.5	26.1	0.584	49.9	17.5	0.054
	Yes	62.9	21.7		55.1	29.3		53.2	29.6		53.7	18.2	
Married	No	62.5	19.9	0.848	60.0	26.7	0.218	53.9	25.8	0.421	52.3	17.4	0.379
	Yes	62.9	20.3		56.1	27.5		51.4	27.4		50.5	17.9	
Obese	No	63.0	19.7	0.729	56.2	27.7	0.493	53.4	27.5	0.150	51.0	17.6	0.633
	Yes	62.2	20.1		58.3	27.2		49.0	25.7		50.0	17.9	
Family history of cancer	No	64.4	19.9	0.011	58.7	28.0	0.028	54.3	28.0	0.002*	51.7	17.5	0.149
	Yes	58.9	20.5		52.6	25.1		46.2	23.5		49.0	18.4	
1st year after cancer diagnosis	No	63.4	20.9	0.542	58.2	28.0	0.358	51.9	27.5	0.954	51.8	17.4	0.331
	Yes	62.2	19.6		55.8	26.7		51.8	26.5		50.1	18.0	
Cancer types	Breast	61.4	20.0	0.161	58.4	28.2	0.278	49.0	26.6	0.073	50.7	19.2	0.564
	Colorectal	65.7	20.3		54.6	24.0		52.7	26.2		50.0	14.8	
	Leukemia	60.9	21.8		56.6	27.4		56.8	25.0		49.1	20.6	
	Lymphoma	67.1	21.6		64.2	28.7		60.8	29.3		54.9	16.1	
	Others	60.5	18.5		54.5	28.8		49.6	27.5		51.2	18.2	
Stage III/IV	I/II	64.0	18.7	0.011	61.8	23.9	0.001*	55.4	25.5	0.001*	53.3	16.0	0.001*
	III/IV	58.4	21.7		50.7	29.4		43.8	26.7		46.5	18.2	
Multiple tumors	No	64.5	19.7	0.003	59.4	26.3	0.004	55.6	26.5	0.001*	52.7	17.1	0.001*
	Yes	58.3	20.7		51.2	28.8		42.6	25.9		46.5	18.4	
Cancer surgery	No	63.4	18.5	0.589	55.7	28.2	0.348	52.4	26.7	0.753	49.7	17.7	0.190
	Yes	62.3	21.4		58.1	26.6		51.6	27.4		52.0	17.8	
Chemotherapy	No	63.9	21.1	0.527	59.2	26.9	0.365	58.2	28.8	0.008	51.6	18.6	0.678
	Yes	62.5	19.9		56.3	27.5		50.1	26.2		50.7	17.5	
Radiation therapy	No	65.3	19.2	0.005	57.6	27.1	0.645	55.1	26.3	0.006	52.4	17.4	0.054
	Yes	59.9	21.0		56.3	27.8		48.0	27.3		49.1	18.1	
Immunotherapy	No	64.5	19.3	0.073	57.4	26.4	0.771	56.9	25.8	0.001*	51.4	17.3	0.615
	Yes	61.1	20.9		56.6	28.4		47.9	27.2		50.6	18.3	
Antibodies	No	63.4	20.4	0.333	57.5	27.5	0.332	53.0	26.1	0.431	51.1	17.5	0.993
	Yes	61.3	19.0		54.5	27.5		50.4	29.6		51.0	19.2	
Metastasis	No	64.9	20.2	0.001*	59.9	26.5	0.001*	56.3	26.2	0.001*	53.6	17.0	0.001*
	Yes	58.1	19.4		50.3	28.2		42.1	26.3		44.8	17.9	
Fever	No	66.4	18.5	0.001*	59.7	26.4	0.010	57.1	26.2	0.001*	54.1	17.8	0.001*
	Yes	57.4	21.4		52.8	28.4		44.1	26.4		46.1	16.6	
Family support	No	59.5	24.8	0.292	57.4	28.2	0.919	54.5	30.3	0.547	48.8	17.0	0.440
	Yes	63.1	19.7		57.0	27.3		51.7	26.7		51.1	17.8	
Chronic disease other than cancer	No	63.3	19.8	0.508	57.5	27.0	0.609	54.3	27.0	0.018	52.9	17.6	0.003
	Yes	62.0	20.8		56.1	28.0		48.1	26.8		47.7	17.6	
Regular exercise	No	60.8	20.7	0.003	53.7	27.4	0.001*	48.2	26.7	0.001*	48.4	17.9	0.001*
	Yes	66.9	18.4		63.8	26.0		59.7	26.1		56.2	16.3	

*The variable is significant using Bonferroni correction cut-off at *α*/*n* = 0.05/24 = 0.0021, where *n* is the number of tests, *P*=P-value.

## Results

### Patients’ Characteristics

Of the 438 cancer patients studied, 64% were female and 28.4% had a family history of cancer. The average age of the sample was 52.9 (±SD = 17.3) with a range of 14–97 years. The median number of months after-cancer diagnoses was 12 (interquartile range 5–24 months). The majority of subjects (76.8%) received chemotherapy, 41.3% had cancer stage III or IV, 30.5% had metastasis, and 24.8% were treated with antibiotics. Other patient characteristics are reported in Table 1.

### Bivariate Analyses

The mean scores by sociodemographics, clinical symptoms, and cancer treatments of each of the eight QoL domains measured by the SF-36 are presented in Tables 1 and 2 and Fig. 1. The mean physical function was 48.30 (±SD = 29.4). According to subgroup analyses, the elderly, those lacking formal education, the unemployed, those diagnosed with stage III/IV, those with metastasis, and those with chronic disease other than cancer have significantly worse physical functions than the other cancer patients. However, regular exercise was predictive of increasing physical function. Higher mean scores of physical functions were found in patients with breast cancer, followed by patients with leukemia, lymphoma, colorectal, and other cancer types.

The mean scale score for role limitations due to physical health was 25.9 (±SD = 37.2). A greater impact on role limitations due to physical health was found in cancer patients with fever. The mean scale score for role limitations due to emotional problems was 31.6 (±SD = 41.4). Cancer patients with stage III or IV, multiple tumors, and fever reported significantly poorer role limitations due to emotional problems when compared to their counterparts. The mean scale score for vitality was 43.9 (±SD = 22.0), and significantly lower scores on vitality were observed in patients with old age, stage III or IV, multiple tumors, metastasis, and fever when compared to their counterparts. However, patients who regularly exercised reported higher scores on vitality than those who did not exercise.

The mean scale score for emotional wellbeing was 62.8 (±SD = 20.2). Lower mean scores on emotional wellbeing was found in cancer patients with metastasis and fever when compared to their counterparts. The mean scale score for social functioning was 57.0 (±SD = 27.4). Cancer patients with stage III or IV and metastasis reported significantly lower scores on social functioning when compared to their counterparts, while patients who practiced regular exercise reported higher scores on social functioning than those who did not. The mean scale score for pain was 52.0 (±SD = 27.0). Bodily pain was significantly increased in patients with a family history of cancer, stage III or IV, multiple tumors, receiving immunotherapy, metastasis, and fever, while patients who regularly exercised reported less bodily pain than those who did not exercise. The mean scale score for general health was 50.9 (±SD = 17.8). Patients with stage III or IV, multiple tumors, metastasis, and fever reported lower scores on general health when compared to their counterparts.

### Regression Analyses

Multivariate analyses (Table 3) showed that elderly and stage III or IV were found to be significant negative predictors of physical health (decrease in physical health scores of 13.79 and 7.82, respectively), while regular exercise was found to be a significant positive predictor of physical health (increase in physical health score of 8.82). A family history of cancer had a negative impact on role limitations due to physical health (decrease in role limitations due to physical health score of 10.3). Patients with chronic disease other than cancer had a positive impact on role limitations due to emotional problems (increase in role limitations due to emotional problem score of 19.66). Poor vitality was predicted by the elderly and those receiving radiation therapy (decrease in vitality scores of 8.11 and 5.92, respectively), while the presence of other chronic diseases and regular exercise were positive predictors of vitality (increase in vitality scores of 5.11 and 5.54, respectively).

**Table 3:** Multiple regression showing predictors of health-related quality of life in patients with cancers

	Physical functioning		Role limitations due to physical health		Role limitations due to emotional problems		Vitality		Emotional wellbeing		Social functioning		Pain		General health	
	*B*	*P*	*B*	*P*	*B*	*P*	*B*	*P*	*B*	*P*	*B*	*P*	*B*	*P*	*B*	*P*
(Constant)	54.04		21.81		41.78		55.70		62.70		77.07		66.46		62.86	
Female gender	−3.72	.412	3.61	.536	−3.84	.556	0.10	.976	1.09	.720	0.73	.860	−1.92	.623	−2.86	.277
Elderly	−13.79	.001*	1.62	.751	−6.04	.289	−8.11	.005*	1.23	.642	−6.88	.059	0.25	.941	−3.67	.110
University	5.29	.184	0.29	.955	3.89	.496	2.97	.306	1.54	.563	−2.94	.419	4.77	.164	1.10	.632
Employed	3.94	.353	2.88	.598	−2.50	.681	1.50	.627	0.63	.824	−4.56	.240	−3.56	.330	0.13	.956
Married	1.61	.701	−8.98	.096	−4.42	.462	−3.31	.278	−2.42	.388	−5.07	.186	−4.82	.182	−3.41	.160
Obese	−1.57	.667	−4.01	.392	−3.17	.544	2.78	.295	1.45	.550	4.14	.215	−1.27	.685	0.73	.729
Family history of cancer	2.64	.478	−10.3	.033*	−6.55	.221	−4.43	.103	−7.54	.003*	−4.90	.152	−4.14	.197	−1.08	.616
1st year after cancer diagnosis	−1.32	.705	6.74	.133	−3.86	.441	−2.77	.276	−5.20	.027*	−7.34	.022*	−4.28	.155	−6.12	.003*
Breast cancer	8.96	.058	10.38	.088	8.91	.189	−4.24	.218	1.31	.679	−0.19	.965	−1.19	.769	−2.33	.393
Colorectal cancer	−1.77	.712	−0.78	.899	2.00	.771	−5.66	.106	2.16	.502	−2.45	.578	0.10	.981	−4.25	.127
Leukemia cancer	4.09	.556	1.19	.895	−5.06	.613	−7.85	.122	−2.52	.589	−8.16	.201	−0.74	.901	−10.2	.012*
Lymphoma Cancer	−0.78	.905	4.90	.562	10.64	.260	0.82	.863	6.54	.137	6.03	.317	9.13	.107	−0.64	.866
Stage III/IV	−7.82	.042*	−5.15	.297	−10.72	.052	−2.89	.302	−0.17	.947	−9.32	.008*	−2.03	.540	−1.63	.463
Multiple tumors	0.43	.918	−5.98	.272	−5.92	.330	−5.07	.100	−4.14	.144	−5.80	.135	−8.08	.027*	−2.35	.338
Cancer surgery	−0.41	.908	4.37	.340	−0.21	.968	−0.51	.844	0.96	.687	−0.99	.762	0.76	.803	0.72	.727
Chemotherapy	−0.56	.902	−2.23	.705	−2.63	.689	−0.91	.786	1.99	.516	−1.56	.710	−3.77	.340	−1.84	.488
Radiation therapy	−6.08	.078	−5.62	.205	0.01	1.000	−5.92	.019*	−8.05	.001*	−0.82	.794	−4.48	.131	−3.78	.059
Immunotherapy	−5.09	.188	−4.06	.414	−5.42	.329	−5.42	.055	−1.76	.496	−0.39	.912	−7.47	.026	0.71	.751
Antibodies	0.32	.935	6.08	.232	−3.58	.528	2.53	.380	−0.41	.875	0.78	.829	3.12	.360	3.02	.187
Metastasis	−5.68	.219	3.55	.551	−1.99	.764	−4.65	.167	−3.05	.324	−4.21	.321	−6.18	.121	−8.34	.002*
Fever	−0.59	.867	−5.74	.205	−8.41	.097	−2.98	.245	−5.54	.019*	−6.15	.057	−8.01	.009*	−4.93	.016*
Family support	5.38	.353	5.75	.441	4.16	.618	6.75	.111	9.70	.013*	2.12	.689	8.82	.078	7.43	.028*
Chronic disease other than cancer	−0.33	.926	7.47	.102	19.66	.001*	5.11	.048*	0.22	.926	2.58	.427	−2.52	.409	−2.64	.199
Regular exercise	8.82	.013*	7.78	.088	9.75	.056	5.54	.032*	2.45	.301	6.66	.041*	5.76	.060	4.97	.016*
Model summary																
*F* value		3.108	1.496		1.957		2.834		2.448		2.450		3.280		3.269	
*P* value		0.001	0.067		0.006		0.001		0.001		0.001		0.001		0.001	
*R* ^2^		0.204	0.110		0.139		0.189		0.168		0.168		0.215		0.212	
*R*		0.452	0.331		0.373		0.435		0.410		0.410		0.464		0.461	

*Adjusting for other predictors in model, predictor is significant at *α* = 0.05. *B* represents the partial regression coefficients. *F* value represents the test value of overall significance of the linear regression model. *R* ^2^ represents the proportion of variance explained. *R* represents multiple correlation coefficients

Family history of cancer, newly diagnosed cancer patients (first-year-after-cancer diagnosis), radiation therapy, and fever were negatively correlated with poor emotional wellbeing (decrease in emotional wellbeing scores of 7.54, 5.20, 8.05, and 5.54, respectively), while family support was positively correlated with better emotional wellbeing (increase in emotional wellbeing score of 9.70). Newly diagnosed cancer patients and stage III or IV were negatively correlated with poor social functioning (decrease in social functioning scores of 7.34 and 9.32, respectively), while regular exercise was positively correlated with better social functioning (increase in social functioning score of 6.66). Cancer stage III or IV and fever had negative impacts on pain (decrease in pain score of 8.08 and 8.01, respectively). Newly diagnosed cancer patients, leukemia patients, those with metastasis, and those with fever had negative impacts on general health (decrease in pain score of 6.12, 10.2, 8.34, and 4.93, respectively), while those with family support and regular exercise regimens had positive impacts on general health (increase in general health scores of 7.43 and 4.97, respectively).

## Discussion

This survey addresses health outcomes in a sample of Saudi Arabians with different types of cancer. There is a lack of research addressing health-related quality of life in patients with different cancers in Saudi Arabia. This study is of interest to QoL researchers and providers caring for cancer patients. It has identified several predictors that appear to be correlated with QoL in cancer patients. One of our findings was that the elderly reported poorer vitality and physical function. These findings are consistent with previous studies in demonstrating older cancer patients may have a negative impact on QoL [5, 6]. It is also evident that patients with first-year-after-cancer diagnosis reported a poorer health-related quality of life. Specifically, patients with first-year-after-cancer diagnosis tended to have poor emotional wellbeing, social function, and general health (decrease in SF-36 scores of 5.20, 7.34, and 6.12, respectively). An Iranian study has also shown that first-year-after-cancer diagnosis is a predictor for poor physical, emotional, and social functioning [24]. Cancer disclosure and patient’s quality of life and its impact on cancer treatment and management must be assessed as their relation has yet to be fully studied in Saudi Arabia.

Our study investigated the association between cancer treatments and QoL. Vitality and emotional wellbeing are reported significantly worse among those who received radiation therapy. Several other studies have also shown that poor QoL is linked with cancer treatments [2–4]. An interventional study is warranted to assess the impact of radiation therapy on QoL.

The study also compares the QoL of survivors with different types of cancer. The QoL depends on the location of the cancer. Leukemia was found to be associated with poor quality of life. This has been frequently addressed in various studies [25–27]. QoL assessment in patients with leukemia can provide insights into the effects of leukemia treatment and its management.

This study also investigated the association between regular exercise and QoL of patients with cancers. Other studies have shown similar findings [28–30]. In our study, exercise tended to improve physical function, role limitations due to emotional problems, vitality, social function, and general health (increase in SF-36 scores of 8.82, 9.75, 5.54, 6.66, and 4.97, respectively). The effectiveness of physical exercise must be assessed in Saudi cancer patients as an intervention to improve QoL and control cancer outcomes. Several limitations were noted. The cross-sectional design may not allow causality assessment. There is a potential for sampling selection bias, in that cancer patients who are attending outpatient clinics may more often be likely to participate, given the perceived severity of their cancer. However, this research has clearly identified several factors that appear to affect QoL in cancer patients.

## Conclusions

Regular exercise in cancer patients was a significant positive predictor of better vitality, social function, and general health. Newly diagnosed cancer patients (first-year-after-cancer diagnosis) tended to experience significantly poor QoL in several domains. The effectiveness of exercise must be assessed in Saudi cancer patients as an intervention to improve QoL.
